# Cutaneous vessel features of sensitive skin and its underlying functions

**DOI:** 10.1111/srt.12819

**Published:** 2019-12-03

**Authors:** Wen‐cai Jiang, Hui Zhang, Yafei Xu, Changqing Jiang, Yingying Xu, Wei Liu, Yimei Tan

**Affiliations:** ^1^ Skin and Cosmetic Research Department Shanghai Dermatology Hospital Shanghai China; ^2^ School of Life Science Fudan University Shanghai China; ^3^ Nursing Department Yangpu Hospital Tongji University Shanghai China; ^4^ Department of Dermatology the General Hospital of Air Force Haidian China

**Keywords:** chemical probes, cutaneous vessels features, dynamic optical coherence tomography, sensitive skin

## Abstract

**Background:**

Following the sufficient studies of the effects of skin barrier impairment and heightened neural reaction on sensitive skin (SS), many scholars have paid great attention to the roles of superficial microvasculature in SS.

**Methods:**

By questionnaire survey, lactic acid sting test, and capsaicin test, eligible subjects were classified as normal skin, only lactic acid sting test positive (LASTP), only capsaicin test positive (CATP), and both positive (both LASTP and CATP). D‐OCT was used to photograph images for evaluating the cutaneous vessels features each group.

**Results:**

Totally 137 subjects completed the study. Compared with LASTN group, the vascular vessels were closer to epidermis in LASTP group. Mesh and branching vessels were more popular in SS than normal skin. High blood vessel density was more prevalent in SS, while low density frequently presented in normal skin. The vascular depth had a closely negative correlation with face flushing and SSS, and vascular shapes had a good positive correlation with face flushing and SSB.

**Conclusions:**

Our study indicates that there is a significant difference in vascular depth, shape, and density between SS and normal skin which is valuable to explore SS pathologic mechanism and to further investigate cutaneous microvasculature functions in SS.

## 1 INTROUDUCTION

Sensitive skin (SS) is characterized by frequently subjective complaints of discomfort to common products such as cosmetics or toiletries, without predictable visible sign of irritation and has a considerable impact on quality of life,^1^ which has drawn great attentions in dermatologists and cosmetic scientists. Many studies have been conducted to investigate influent factors and etiology of SS in the past decades. At present, the declined barrier function resulting in an increasing permeability of the stratum corneum has been recognized as a crucial mechanism.^2^, ^3^, ^4^ Besides that, other pathogeneses have also been reported such as the heightened vascular and neural reactions.^5^, ^6^, ^7^, ^8^ Although the exact mechanism of SS still remains poor understanding, epidemiologic investigations indicated that most of self‐perceived sensitive skin (SPSS) claimed more frequent face flushing relative to non‐sensitive skin, when climate changes, or they have unstable emotions or unknown reasons.^9^, ^10^, ^11^ Moreover, skin redness as one common symptom had been debated identified in an official definition of sensitive skin established by the International Forum for the Study of Itch (IFSI) through the IFSI special interest group (SIG).^12^ Owing to part of SS accompanied by skin disorders such as contact dermatitis, AD, or rosacea, Yamaski proposed that flushing or blushing erythema may be due to chronic inflammation derived from innate immune system.^13^ Actually, most SS subjects had no cutaneous disorders during the study. Obviously, other factors may be involved in face flushing such as vascular component variations that had been demonstrated by Seidenari 14 and Roussaki‐Schulze.^15^ Recently, a systematical analysis on 27 out of 1701 papers of SS made by Richters who concludes that the increasing vascular reactivity is closely associated with SS.^16^ Our team has also paid serious attention to SS and noted that there was no difference in cutaneous blood flow between SS and normal skin in previous studies 17, 18 and therefore speculates whether superficial cutaneous vessel features are potential factors of SS face flushing.

Dynamic optical coherence tomography (D‐OCT) is an emerging technology in dermatology, which provides both functional (eg, blood flow data) and morphologic information about the skin microvasculature, therefore has been used in the diagnosis and prognosis of cutaneous tumors 19, 20 and other skin conditions.^21^, ^22^ D‐OCT has recently been studied in normal skin to provide a frame of reference on the qualitative features of vascular morphology.^23^ In another study, more vascular features were defined clearly, and it was helpful to investigate the function of cutaneous vessels more comprehensively and objectively 24.

In this study, the aim of our team was to investigate the cutaneous vessel features of SS and its relationship with SS. The study procedure is that volunteers are screened and classified by questionnaire survey, lactic acid sting test (LAST), and capsaicin test (CAT), and their D‐OCT images are collected for evaluating vascular features including maximal blood flow signals (MBFS) and its corresponding vascular depth, shape, distribution, and density.

## 2 MAETERIALS AND METHODS

### 2.1 Participants

The study was conducted in Shanghai, China, from December 2018 to February 2019. A total of 137 qualified volunteers aged from 18 to 60 years old were enrolled to complete the study, who were divided into four groups according to questionnaire survey, LAST, and CAT. Those who have active skin diseases (eczema, acne, seborrheic dermatitis, etc) were excluded. The study was approved by the Ethics Committee of Shanghai Skin Diseases Hospital, and the written informed consents from all subjects were obtained prior to the study.

### 2.2 Questionnaire survey

The questionnaire consisted of two parts as following: to self evaluate whether to be sensitive skin and related influent factors of SS.^9^, ^24^, ^25^ The latter include 10 questions: whether you experienced frequent flushing for no apparent reason, you had discomfortable sensation when environment changes, climatic shift, or emotion stimulus, whether you had a history of cosmetic allergy or experienced frequent unpleasant sensation after using cosmetics, whether you suffered from AD or eczema as a child or had a family history of allergic skin diseases, and whether you were a sensitive scalp and had a history of food allergy. Through the questionnaire survey, all subjects were divided into two groups, namely SPSS and non–self‐perceived sensitive skin (NSPSS).

### 2.3 Lactic acid sting test

The volume of a 50 μL 10% lactic acid aqueous solution (Sigma Chemical) was applied to nasolabial fold by single layer of filter paper (8 mm diameters), and the same volume of distilled water was applied to the other side as the negative control. The test was performed in single blind. The sting scores were recorded at three special time point (30 seconds, 2.5 minutes, and 5 minutes) using a 4‐point scale (0 = none of sting, 1 = slight sting, 2 = moderate sting, and 3 = severe sting). If the sum scores of sting at 2.5 and 5 minutes were ≥3 on the side of the lactic acid, the subject was defined as a positive one (LASTP), otherwise, as a negative (LASTN). The sum scores of sting (SSS) were recorded at three time points.

### 2.4 Capsaicin test

Based on the results of preliminary study, 1.0 × 10^−5^ capsaicin aqueous solution (*w*/*v*) was applied in the study. This solution was prepared from pure‐grade capsaicin powder (8‐methyl‐N‐vanillyl‐6‐none‐namide ≥98.0%, Haida Chemical Co.) in 10% ethanol solution which was obtained using absolute ethanol (99.85%, Tiangen Biochemical Technology Co.) and distilled water (*v*/*v*). The test procedures listed as follows 25: Firstly, the 10% ethanol solution was applied on the nasolabial folds with single‐use cotton swab for two minutes. If the subjects did not report any discomfort, the test continued. The single layer of filter paper with a 0.8 cm diameter was wetted with 50 μL 1 × 10^−5^, capsaicin solution was randomly placed on each side of nasolabial fold, and the 10% ethanol solution was done on the other side. The subject was asked to report any abnormal sensation and its severity at the three special time points (30 seconds, 2.5 minutes, and 5 minutes) using the following scoring scale: 0 = none of perception, 1 = barely perceptible, 2 = slightly perceptible, 3 = moderately perceptible, 4 = strongly perceptible, and 5 = painful. If the capsaicin side felt burning that lasted more than 30 seconds with a degree ≥3, the capsaicin test was positive (CATP), otherwise the capsaicin test was negative (CAPN). Meanwhile, the sum scores of burning (SSB) were calculated at three time points.

According to above tests, subjects were classified as normal skin and SS. The former must meet simultaneously three criteria: NSPSS, LASTN, and CAPN, and the latter must be a SPSS subject who was at least LASTP or CATP as well. Other than that, the others were excluded from the study.

### 2.5 Dynamic optical coherence tomography imaging

D‐OCT images were acquired using the hand‐held probe OCT (VivoSight t^®^ Dx Scientific, Michelson Diagnostics Ltd.) which provides a 6mm × 6 mm field of view. The system can show vertical and horizontal plane images of each target area at any desired depth. En‐face images were used to assess vascular characteristics.

In the study, D‐OCT scan was performed at the intersection of the horizontal extending line of nose tip and the outer corner of eye on left or right cheek, which provided cutaneous blood flow signal values at a depth of 1 mm with a 50 μm steps. Our previous study showed cutaneous blood flow signals change like an inverted letter “V” as increasing skin depth. That means MBFS reflects more microvasculature information. Here, we were interested only in MBFS and its corresponding vascular patterns (shape, distribution, and density) and depth (the depth is calculated automatically by D‐OCT own software). All OCT images were read by two trained dermatologists who were blinded to subjects grouping. If a serious disagreement on reading result happens between them both, the final conclusion was determined by the senior expert.

All of the tests were conducted under controlled humidity (40%‐60%) and temperature conditions (20‐22°C), and the intervals of each test were at least 1 week.

### 2.6 Statistical analysis

Statistical analysis was carried out using SPSS software (SPSS version 17.0). The mean and standard deviation were calculated for the quantitative variables, and the qualitative variables were expressed as frequency. Several comparative studies were performed using One‐way ANOVA for the quantitative variables and chi‐square test (*χ* ^2^ test) for the categorical variables. Spearman's correlation coefficients were calculated and expressed as *r*. The level of significance was set at 5.0%.

## 3 RESULTS

### 3.1 Distribution of skin sensitivity

According to the questionnaire, LAST, and CAT, all participants were divided into four groups. The details were listed in Table 1. There was no significant difference in sex ratio or age among four groups (*P* > .05).

**Table 1:** Demographic data of each group

Questionnaire	LAST/CAT	Sex (Male/Female)	Age (y)
NSPSS	LASTN‐CATN (n = 39)	19/20	42.8 ± 9.4
SPSS	LASTN‐CATP (n = 32)	14/18	43.4 ± 9.5
	LASTP‐CATN (n = 32)	14/18	42.5 ± 9.8
	LASTP‐CATP (n = 34)	15/19	41.7 ± 9.0

### 3.2 Maximal blood flow signals and vascular depth

**Figure 1:**
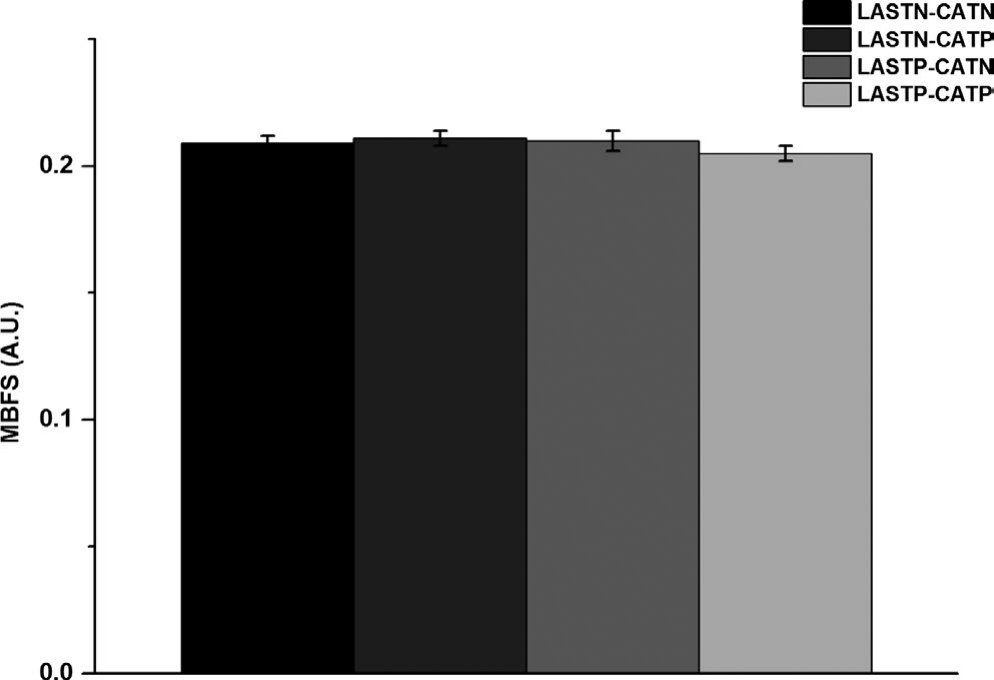
Comparison of MBFS values among four groups (mean ± SD)

**Figure 2:**
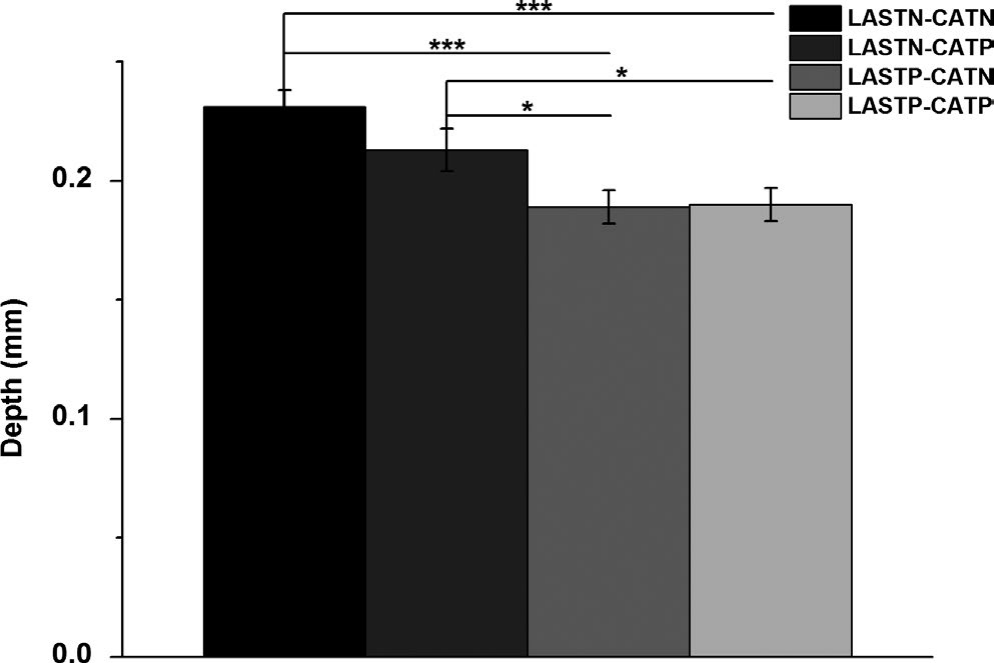
Comparison of vascular depth corresponding MBFS among four groups. The blood vessels of LASTN groups were deeper than that of LASTP (mean ± SD, ^*^ *P* < .05, ^***^ *P* < .001)

As shown in the Figure 1, MBFS values were (0.209 ± 0.003) in both LASTN and CATN group, (0.211 ± 0.003) in only CATP group, (0.210 ± 0.004) in only LASTP group, and (0.205 ± 0.003) in both LASTP and CATP group, without significant difference among four groups (*P* > .05). Its corresponding vascular depth were (0.231 ± 0.007) mm, (0.213 ± 0.009) mm, (0.189 ± 0.007) mm, and (0.190 ± 0.007) mm, respectively (Figure 2). The blood vessels of both LASTP and CATP and only LASTP were closer to epidermis than that of both LASTN and CATN (*P* < .001) or only CATP (*P* < .05).

### 3.3 Vascular pattern

**Figure 3:**
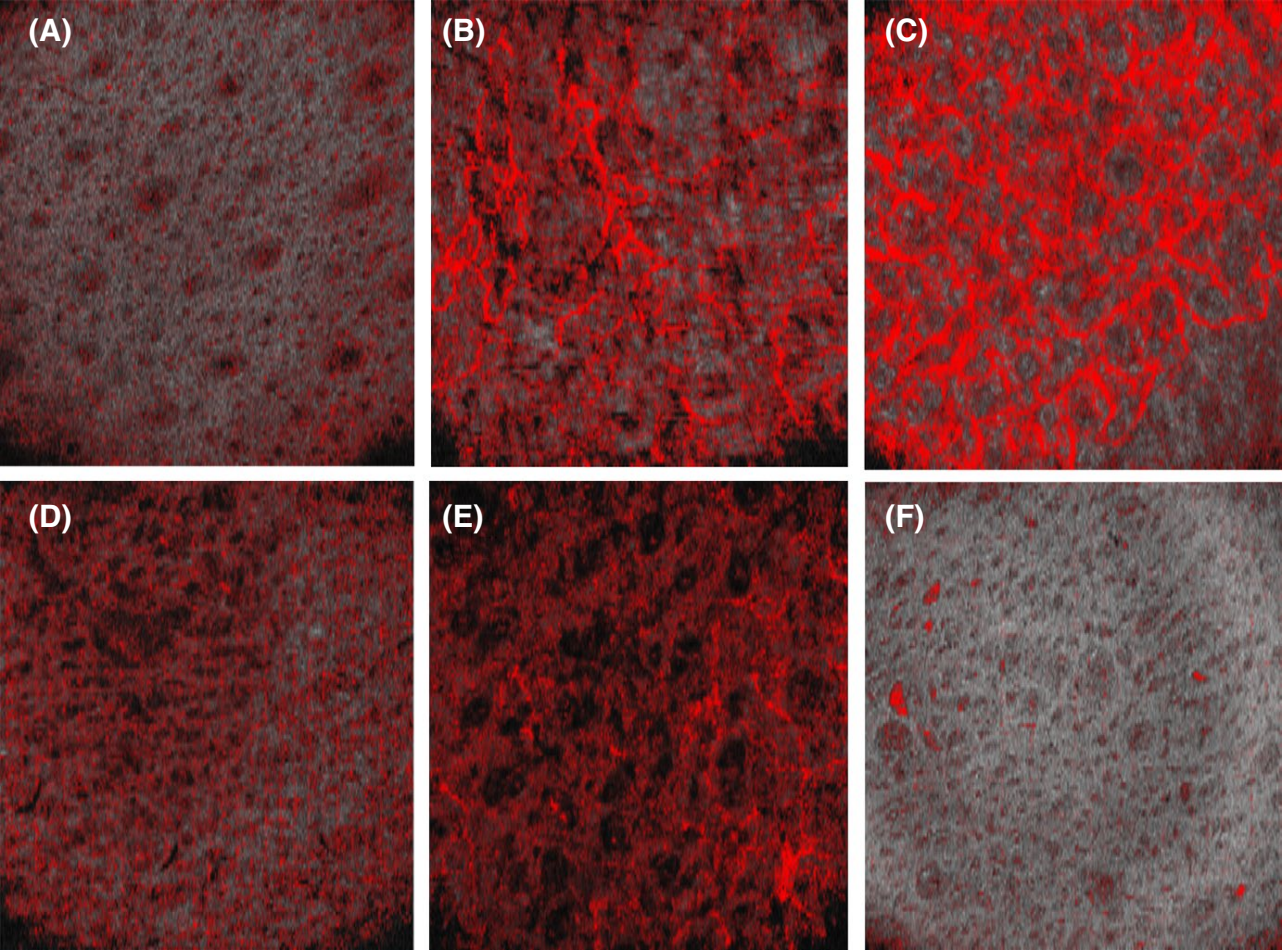
Vascular pattern corresponding to MBFS in en‐face images of D‐OCT. Vascular shapes included “mottle” (A) and (D), “branching” (B), and “mesh” (C). Distribution patterns were presented as “regular” (C) and (D), “irregular” (A), (B), and (E), and “clustered” (F). Vascular density had three grades, low: (A) and (F), moderate: (E), and high: (B), (C), and (D). A, comes from the subject No. 11 who is both LASTN and CATN groups. B and D, come from the subject No. 114 and 93 who are both LASTP and CATP groups. C and E, come from the subject No. 74 and 130 who are only LASTP group. F, come from the subject No. 59 who is only CATP group [Colour figure can be viewed at wileyonlinelibrary.com]

The details of vascular pattern were listed in Table 2. According to the analysis of all en‐face OCT images, vascular shapes were classified as three categories as following: mottle, mesh, and branching (Figure 3). Among them, the mottle was most common shape in each group, with a highest frequency of 74.4% in both LASTN and CATN. The highest frequency of branching vessels presented in both LASTP and CATP, up to 32.2%, and mesh shape presented a highest proportion of 34.4% in only CATP. There was a significant difference in frequency among four groups (*χ* ^2^ = 14.119, *P* = .028), while there was no statistical difference among three subgroups of SPSS (*χ* ^2^ = 5.698, *P* = .223).

**Table 2:** The number and proportions of vascular patterns in each group (n [%])

Groups	Shape			Distribution			Density		
	Mottle	Branching	Mesh	Regular	Irregular	Clustered	Low	Moderate	High
LASTN‐CATN	29 (74.4)	6 (15.4)	4 (10.2)	13 (33.3)	15 (38.5)	11 (28.2)	13 (33.3)	20 (51.3)	6 (15.4)
LASTN‐CATP	14 (43.8)	7 (21.8)	11 (34.4)	9 (28.1)	15 (46.9)	8 (25.0)	4 (12.5)	20 (62.5)	8 (25.0)
LASTP‐CATN	19 (59.4)	6 (18.8)	7 (21.8)	11 (34.4)	10 (31.2)	11 (34.4)	4 (12.5)	16 (50.0)	12 (37.5)
LASTP‐CATP	12 (35.2)	11 (32.4)	11 (32.4)	8 (23.5)	11 (32.4)	15 (44.1)	5 (14.7)	17 (50.0)	12 (35.3)

The distribution of vessels included regular, irregular, and clustered depending on vascular pattern and aspect throughout the imaged area (Figure 3). In the study, a total of 29.9% images presented as regular, 37.2% as irregular, and 32.9% as clustered. And no statistical difference was found in frequency of vessel distributions among four groups (*χ* ^2^ = 4.396, *P* = .623), and there was no statistical difference between SS and normal skin (*χ* ^2^ = 0.591, *P* = .744).

Referring to Ulrich's grading standard,^26^ the vascular density was graded into low, moderate, and high levels (Figure 3). The moderate blood vessel density was predominant accounting for over 50% in total. Among four groups, there was no significant difference in frequencies of different blood vessel density (*χ* ^2^ = 10.681, *P* = .099). However, the proportions of high blood vessel density in SS groups were approximate twice as high as that of normal skin, while that was opposite in low density. And there was a significant difference between SS and normal skin (*χ* ^2^ = 14.422, *P* < .001).

### 3.4 Correlation between vascular features and SS

Questionnaire survey indicated there were significant differences in frequent face flushing without reason (*P* < .01), environment changes (*P* < .05), climatic shift (*P* < .01), a history of cosmetic allergy (*P* < .001), frequent cosmetic stimulus experiences (*P* < .05), and a history of AD or eczema as a child (*P* < .001) among four groups. The correlation was calculated between the above factors and vascular depth and shapes. As is shown in Table 3, there was a close negative correlation of vascular depth with climatic shift (*P* < .001), face flushing (*P* < .001) and SSS (*P* < .001), and vascular shapes had a positive relationship with face flushing (*P* < .001) and SSB (*P* < .01).

**Table 3:** The correlation of vascular pattern with related factors of SS

		Environment change	Climatic shift	Flushing	Cosmetic allergy	Cosmetic stimulus	AD or eczema history	SSS	SSB
Depth	*r*	−.118	−.224*	−.577*	−.009	−.041	.033	−.394*	−.164
	*P*	.171	.004	.000	.920	.634	.699	.000	.055
Shape	*r*	−.016	.123	.481*	.089	.073	.033	.14	.256*
	*P*	.851	.152	.000	.302	.396	.705	.102	.003

Abbreviations: SSB, sum scores of burning; SSS, sum scores of sting.

* Correlation is significant at the .01 level (two‐tailed).

## 4 DISCUSSION

Since SS seems to be a multi‐dimensional condition, precise identification and categories are of high importance to unravel its pathomechanism. As a classic method, self‐assessment questionnaire survey has been used extensively to identify SS,^11^, ^16^ while cannot define SS subtype. Capsaicin, as a nature agonist of the transient receptor potential vanilloid subfamily, member 1 (TRPV1), is expressed in keratinocytes and peripheral sensory nerve fiber,^27^ which is suggested to be a reliable tool to screen SS with heightened neuroreactivity.^10^, ^28^ Sodium lauryl sulfxate (SLS) has proved highly practical and reliable for skin barrier function assessment.^7^, ^29^ However, it is not applicable to be tested on the face. Most existing studies have shown that facial skin could be more sensitive relative to other bodies.^30^, ^31^ So, the SLS results from other bodies could not represent facial skin barrier. LAST is proposed as the best predictor available for SS,^32^ and the existed studies have shown higher transepidermal water loss in LASTP.^8^, ^33^ Namely, LASTP was equal to a SS with impaired skin barrier in the study. Therefore, only LASTP, only CATP, and both LASTP and CATP represented, respectively, SS with defective barrier function, heightened neural reaction, and a mixed in the present study. Among four groups, differences in vascular features are fully attributed to sensitive skin due to a good consistency of sex ratio, age, and measurement site.

The cutaneous microcirculation is organized into two horizontal dermal plexuses linked by communicating vessels.^34^ An upper horizontal network that is contained in the papillary dermis 1 to 2 mm below the epidermal surface plays a role in supplying nutrition, oxygen, and heat regulation for epidermis. Thus, the MBFS at an average depth of (0.21 ± 0.046) mm is considered to represent only the superficial skin blood flow in the study and its corresponding blood vessels play a great role in epidermal functions. Similar to cutaneous blood flow reported in previous studies,^17^, ^18^, ^35^ MBFS is not different between four groups. It means that the blood flow of superficial microvasculature is not a key risk factor of SS.

The vascular depth corresponding to MBFS was distinctly different among four groups, and the average depth was 0.231 ± 0.007 mm in both LASTN and CATN group, which was significantly deeper than that of only LASTP and both LASTP and CATP. It may be relative to thinner epidermal thickness in SS than normal skin.^36^ Interestingly, the vascular depth of only CATP group, being close to that of both LASTN and CATN, was also significant difference from only LASTP and both LASTP and CATP. We speculate that superficial microvessels in LASTP have lied in closer proximity to epidermis that is a physiologic compensatory, as well observed in psoriatic lesion skin, due to skin barrier impairment.^37^ Additionally, more superficial vessels suffer easily from external stimuli and result in vascular hyperreaction and inflammatory mediators release, which is proposed one of SS mechanisms.^32^, ^38^ In the study, what supported the conclusion was a strongly negative correlation between cutaneous vascular depth with both frequent face flushing and SSS. And SSS had been reported to have a positive correlation with the level of barrier impairment.^39^ Therefore, it is suggested that vascular depth is a valuable predictor of SS with skin barrier dysfunction.

Linds Andersen suggested the differences in vascular shapes depended on anatomic locations and depth in normal tissue.^23^, ^40^ In the present study, blood vessels lie in a depth from 0.189 mm to 0.231 mm, and the proportion of three types of vascular shapes was significantly different among four groups. The predominant shape was mottle that was presented in between 35.2% and 74.4% of subjects, and mesh and branching were observed primarily in SS with CATP. Mesh is interpreted as vascular plexuses,^23^ compared with mottle vessels in the condition of same MBFS that can support more oxygen and nutrition to result in peripheral nerves faster conduction velocity and more serious reactions to external stimulus.^41^ The present study showed subjects with mesh and branching vessels were more sensitive to capsaicin, and a positive correlation was found between vascular shape and SSB. At the same time, vascular shape also was strongly positive correlation with face flushing. According to the results, it is suggested vascular shape is one of predisposing factors of SS, especially to CATP.

In the study, there was no significant difference in frequency of vascular distribution among four groups, and the irregular distribution was more prevalent in LASTN group, while a clustered was frequent in LASTP group. So, it is interesting to further study whether barrier dysfunction plays roles in vascular distribution. In addition, the reasons the vascular distribution in the study was different from Lindsø Andersen's study^23^ may be come from the difference in study population, anatomic location, target depth, and evaluation standard of vascular distribution.

There was no statistical difference in vessel density among four groups either. Among them, the moderate blood vessel density was most prevalent accounted for over 50% of subjects. In SS group, the high blood vessel density in frequency was over twice than low blood vessel density, while the frequencies of both is opposite in normal skin. Many studies into mechanisms underlying microvascular proliferation have recognized the involvement of several angiogenic factors from mainly keratinocytes of lesion skin in psoriasis.^42^, ^43^, ^44^ But, there is now no research on expression of angiogenic factors in SS. Nevertheless, it is no doubt that blood vessel density is a promising means of predicting SS.

In conclusion, our study finds significant differences in vascular depth, shapes, and density between SS and normal skin. And vascular depth has a closely negative correlation with face flushing and SSS, and vascular shapes have a good positive correlation with face flushing and SSB. Therefore, superficial vascular features are considered as valuable predictors and potential predisposing factors of SS, whether they work directly or indirectly.

However, SS is not limited to face; additional study is therefore needed to collect cutaneous vessel features of other anatomic locations so as to better understand its influence on SS. In addition, part of cutaneous vessel features seems to be reasonably interpreted as risk factors of SS. But, as most of case‐control studies, there is a limitation that is no strong chronologically reasonable evidence of causality. In other words, does the difference of vascular features induce SS or derives from SS? To figure out the question, we are going to categorize all OCT images in our database by the above vascular features and observe the distribution of SS in the following study. If necessary, another study will also be performed to observed vascular features before and after SS treatments. In summary, great efforts should be made to clarify the function of cutaneous microvasculature in SS.
